# Splicing factor HNRNPD and alternative splicing of MAP4K4 are associated with cell apoptosis and immune microenvironment features in Wilms’ tumor

**DOI:** 10.1371/journal.pone.0354712

**Published:** 2026-08-06

**Authors:** Chunlei Yang, Man Liao, Haolun Xu, Jun Wang, Haibin Bao, Man Zhang, Can Li, Tian Zheng, Gang Li

**Affiliations:** 1 Department of Urology, Wuhan Children’s Hospital, Tongji Medical College, Huazhong University of Science and Technology, Wuhan, China; 2 Department of Women’s Health Care, Wuhan Children’s Hospital, Tongji Medical College, Huazhong University of Science and Technology, Wuhan, China; 3 Department of General Surgery II (West Campus), Wuhan Children’s Hospital, Tongji Medical College, Huazhong University of Science and Technology, Wuhan, China; 4 Department of Ophthalmology, Zhongnan Hospital of Wuhan University, Wuhan, China; Centre de Recherche en Biologie cellulaire de Montpellier, FRANCE

## Abstract

**Background:**

As the most common malignant renal tumor in children, the progression of Wilms’ tumor is frequently driven by abnormal alternative splicing (AS), cell death imbalance, and an immunosuppressive microenvironment. However, the precise regulatory chain connecting these three critical elements remains largely unexplored. This study aimed to systematically construct and characterize an “AS-cell death-immunity” regulatory network in Wilms’ tumor.

**Methods:**

We performed a comprehensive *in silico* analysis using matched paired Wilms’ tumor and adjacent normal RNA-seq data from the GSE138869 cohort. The SUVA algorithm was employed to identify cell death-related regulated alternative splicing events (RASEs). A tripartite regulatory network was constructed via correlation analysis to link these RASEs with upstream differentially expressed splicing factors (DESFs). Immune cell infiltration was quantified using CIBERSORT. Finally, the *HNRNPD* knockout and FLASH-seq multi-omics dataset (GSE212767) was utilized to computationally validate the predicted regulatory axis.

**Results:**

Our analysis identified 118 cell death-related host genes undergoing significant alternative splicing in Wilms’ tumor. Network integration highlighted a critical regulatory axis where the overexpressed splicing factor *HNRNPD* is strongly correlated with an aberrant AS event (clualt5p51764) in the apoptosis-related kinase *MAP4K4*. Further immune deconvolution demonstrated that both *HNRNPD* upregulation and the *MAP4K4* splicing shift were significantly correlated with increased monocyte infiltration in the tumor microenvironment. Moreover, cross-validation utilizing the GSE212767 dataset confirmed that *HNRNPD* perturbation directly alters *MAP4K4* splicing.

**Conclusions:**

Our computational framework proposes that the *HNRNPD*-*MAP4K4* splicing axis links apoptotic dysregulation to immune microenvironment remodeling in Wilms’ tumor. These correlative *in silico* findings provide a robust, hypothesis-generating basis for discovering novel prognostic biomarkers and developing targeted therapeutic strategies directed at the splicing machinery.

## Introduction

Wilms’ tumor is the most common malignant renal tumor in children, accounting for over 90% of pediatric renal tumors [1]. As an embryonal tumor, it originates from the metanephric blastema and is characterized by a triphasic histological pattern composed of blastemal, stromal, and epithelial components [2]. Wilms’ tumor exhibits distinct histological subtypes, including favorable histology and anaplastic histology [3]. The pathogenesis involves multiple factors, with genetic predisposition playing a crucial role. It is associated with congenital syndromes, oncogene and tumor suppressor gene dysregulation, long non-coding RNA and microRNA aberrations, and chromosomal abnormalities [4,5]. Current treatment mainly consists of a multimodal approach, including surgery, chemotherapy, and radiotherapy, with emerging therapies such as immunotherapy and targeted therapy also being explored [6]. Further research on this disease is of great significance, as although survival rates exceed 90% in developed countries, they are lower in developing countries. Additionally, issues such as recurrence (approximately 15%), long-term treatment side effects (e.g., secondary tumors), and poor prognosis for high-risk subtypes exist [6].

Alternative splicing (AS) refers to the process by which a single gene’s pre-mRNA can be spliced in different ways to produce multiple mRNA isoforms, which in turn can be translated into proteins with different functions [7,8]. AS is regulated by a variety of splicing factors (SFs) and plays a crucial role in numerous biological processes, including cell function, differentiation, development, and disease onset [7,8]. In tumor cells, mutations or abnormal expression of certain SFs can alter the splicing patterns of key genes, thereby promoting tumor proliferation, invasion, and drug resistance. Moreover, AS may also affect processes such as tumor cell metabolism, signal transduction, and immune evasion [9,10]. One study has analyzed the transcriptomic data of high-risk Wilms’ tumor and observed significant differences in AS among tumors from different regions. The splicing variants of the differentially spliced genes are associated with epithelial-mesenchymal transition (EMT), tumor invasion, and muscle-specific phenotypes [11]. Currently, research on the specific mechanisms and potential therapeutic targets of AS in Wilms’ tumor is still in the exploratory stage.

Cell death, which eliminates senescent, damaged, infected, and abnormal cells, is crucial for maintaining homeostasis during development [12]. It plays a complex and key role in tumor development, progression, treatment response, and the immune microenvironment [13]. Targeting the regulation of cell death in combination with an understanding of TME remodeling is an important direction for developing tumor treatment strategies, with high-throughput sequencing technologies providing support for exploring relevant targets [14]. Although apoptosis is non-immunogenic, it can reshape the tumor microenvironment (TME) by releasing signals. In Wilms’ tumor, several studies have revealed the regulation of apoptosis by non-coding RNAs and their clinical significance [15,16]. Wilms tumor suppressor peptide (WTSP) can induce tumor cell apoptosis and necrosis by downregulating BCL-2 and upregulating BAX. It can also inhibit proliferation by downregulating β-catenin [17]. These mechanisms indicate that the imbalance of the apoptosis pathway is a crucial aspect in the development and progression of Wilms’ tumor.

**Fig 1:**
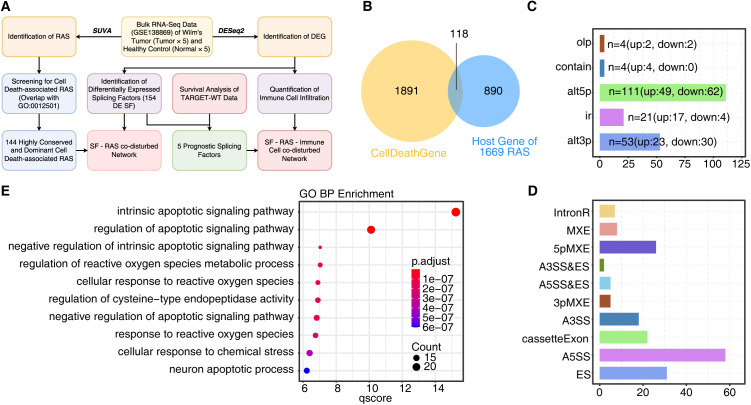
Landscape of RAS in Wilms’ tumor and its association with cell death. (A) A flowchart illustrating the overall research design, including data acquisition, bioinformatic analyses, and key findings of the study. Public dataset identifiers are included for reference. (B) Venn diagram showing the overlap between genes associated with cell death pathways and genes exhibiting RASEs identified in the Wilms’ tumor cohort. (C) Number of cell death-related RASEs identified in the GSE138869 dataset using the SUVA algorithm. (D) Number of SUVA-detected cell death-related RASEs that were annotated to canonical ASE types. (E) GO-BP enrichment analysis of the cell death-related RASGs.

All studies have emphasized the presence of an immunosuppressive microenvironment in Wilms’ tumor [17]. The activation or dysregulation of cell death pathways can directly affect the composition of the immune microenvironment in Wilms’ tumor. Research on the interplay between cell death and the immune microenvironment in Wilms’ tumor remains limited, with a particular lack of exploration into the role of AS in this process. We investigated the regulatory links among these three factors by downloading and analyzing a transcriptomic dataset related to Wilms’ tumor. First, we used the AS analysis software SUVA to identify and filter cell death-related regulated alternative splicing (RAS) events between control and tumor samples. Differential expression of SFs between the two groups was identified by DESeq2. Subsequently, based on the correlation analysis of RAS and differentially expressed SFs (DESFs), we constructed an SFs-RAS co-disturbance network. And based on the TARGET-WT data, we performed prognostic analysis on the 5 significant SFs. Further, through Cibersort-based quantification of immune infiltration, we analyzed the correlation between the above-mentioned SFs and RAS events (RASEs) and the proportion of immune cells, and constructed an SFs-RAS-immune cell co-disturbance network. The specific analysis process is shown in Fig 1A. Finally, we focused on the potential regulatory effect of the splicing factor HNRNPD on the AS of *MAP4K4*, which may regulate the apoptosis of Wilms’ tumor cells and is associated with immune cell infiltration and may be involved in the regulation of the immune microenvironment. These findings, though requiring further experimental validation to confirm causality and clinical relevance, provide a novel direction for identifying prognostic biomarkers and developing precise therapeutic strategies targeting splicing regulation in Wilms’ tumor.

## Materials and methods

### Retrieval and process of public data

RNA sequencing (RNA-seq) data and associated clinical information for Wilms’ tumor were retrieved from the Gene Expression Omnibus (GEO) database (accession number: GSE138869) on 27/09/2025. Additionally, clinical metadata and pre-processed gene expression profiles from the TARGET-WT (Therapeutically Applicable Research to Generate Effective Treatments – Wilms Tumor) cohort were utilized. TARGET-WT is a large-scale, high-quality reference consortium specifically established to identify genomic alterations and therapeutic targets in pediatric renal tumors. Due to its substantial sample size, this cohort serves as an ideal validation database for evaluating the prognostic significance of our candidate genes in clinical outcomes. For validation purposes, *HNRNPD* knockout RNA-seq and HNRNPD FLASH-seq datasets from HEK293T cells were also acquired from GEO (accession number: GSE212767) on 27/09/2025. Raw data were downloaded from the Sequence Read Archive (SRA). SRA Run files were converted to fastq format with NCBI SRA Tool fastq-dump. The raw reads were trimmed of low-quality bases using a FASTX-Toolkit (v.0.0.13; http://hannonlab.cshl.edu/fastx_toolkit/). Then the clean reads were evaluated using FastQC (http://www.bioinformatics.babraham.ac.uk/projects/fastqc).

### Splicing analysis using SUVA

The SUVA (Splicing Usage Variation Analysis) [18] algorithm was employed to detect and quantify all alternative splicing events (ASEs) from the RNA-seq data. To capture comprehensive global splicing alterations, the samples were initially analyzed as two pooled groups (Tumor vs. Normal). Splicing site usage variations were calculated between the cohorts, and specific events were filtered based on differential splicing ratios. Specifically, SUVA can categorize ASEs into five types: alt3p, alt5p, olp, contain, and ir (Fig S1A in S1 File). ASEs with a proportion of SUVA ASEs Ratio (pSAR) > 50%, an absolute differential splicing ratio > 0.15, and a P-value < 0.05 were selected for downstream analysis, which are designated as Regulated Alternative Splicing Events (RASEs). To identify RASEs associated with cell death, the analysis was focused on a pre-compiled list of 2,009 cell death-associated genes. This comprehensive list was obtained by retrieving all human genes annotated to the Gene Ontology term GO:0012501 (programmed cell death), strictly inclusive of all its hierarchical child terms (e.g., apoptotic process, necroptotic process, etc.) from the GO database. The Sogen software was used for the visualization of read distributions and splicing structures of specific ASEs.

### Co-expression analysis of differentially expressed SFs and RAS ratio

The software DEseq2 [19], which is specifically used to analyze the differential expression of genes, was applied to screen the raw count data for differentially expressed genes (DEGs). The results were analyzed based on the fold change (FC ≥ 2 or ≤ 0.5) and false discovery rate (FDR ≤ 0.05) to determine whether a gene was differentially expressed.

### Functional enrichment analysis

To identify functional categories of genes, we employed the clusterProfiler package (v4.6.2) [20], which enabled us to determine Gene Ontology (GO) terms and KEGG pathways.

### Splicing factor binding motif analysis

The RBPBench (https://github.com/michauhl/RBPBench) tool was used to predict the binding motifs of key SFs on specific RASEs. This analysis helped to infer the potential direct regulatory relationship between a SF and a target splicing event.

### Co-expression analysis of splicing ratio of RAS and expression of differentially expressed SFs

Then expression profiles of differentially expressed splicing factors (DESFs) were filtered out from all DEGs according to a catalogue of 603 human SFs retrieved from previous reports [21–24]. The complete list of these 603 SFs used for filtering is provided in S4 Table. To investigate the potential regulatory relationships, a co-disturbed network between the expression levels of DESFs and the splicing ratios of RAS events was constructed. The pairwise Spearman correlation coefficients were calculated using the R base function cor.test. The regulatory pairs were strictly filtered based on the following pre-defined parameter thresholds: (1) host gene FPKM > 3; (2) |differential splicing ratio| > 0.5 between tumor and normal groups; (3) Spearman’s correlation coefficient |r| >= 0.95; and (4) statistical significance P <= 0.01. Finally, the eligible SF-RAS regulatory relationships, along with their associated Gene Ontology Biological Process (GO-BP) terms, were imported into Cytoscape software (version 3.10.1) to visualize the tripartite regulatory network.

### Immune-cell deconvolution from bulk RNA-seq

To evaluate the composition of the tumor microenvironment, we utilized CIBERSORT, a computational deconvolution algorithm that estimates the relative proportions of specific cell types within a complex tissue mixture based on bulk gene expression data. Specifically, we applied the LM22 leukocyte gene signature matrix, which contains 547 genes distinguishing 22 mature human hematopoietic populations, to infer the relative infiltration fractions of these immune cells in our samples (1,000 permutations; quantile normalization disabled) [25]. Cell types with deconvolution P ≤ 0.05 in ≥80% of samples were retained. Statistical significance for immune cell infiltration fractions was calculated using the Wilcoxon rank-sum test. Spearman correlations between immune-cell fractions and splicing ratios were computed across samples.

### Other statistical analysis

Principal component analysis (PCA) analysis was performed by R package factoextra (https://cloud.r-project.org/package=factoextra) to show the clustering of samples with the first two components. After normalizing the reads by TPM (Tags Per Million) of each gene in samples, in house-script (sogen) was used for visualization of next-generation sequence data and genomic annotations. The pheatmap package (https://cran.r-project.org/web/packages/pheatmap/index.html) in R was used to perform the clustering based on Euclidean distance.

### Ethics statement

Because this study is strictly computational and relies exclusively on publicly available datasets (GSE138869 and GSE212767) without involving new human subjects or animal models, additional ethical approval from an institutional review board was not required.

## Results

### Identification and characterization of differential alternative splicing events of cell death-related genes in Wilms’ tumor

To thoroughly investigate the AS patterns of cell death-related genes and the regulatory differences in AS within Wilms’ tumor, we analyzed the publicly available transcriptomic dataset GSE138869 (5 tumor-normal tissue pairs). Using the AS analysis software SUVA (Fig S1A in S1 File), we comprehensively compare the differences in ASEs between the two groups of samples (Fig S1B in S1 File). A total of 1,669 differential ASEs were identified, with alt5p and alt3p being the predominant types of RASEs (Fig S1C in S1 File). Mapping SUVA-identified splicing events to classical AS types revealed A5SS and ES as the most differentially RASEs in tumors (Fig S1D in S1 File), indicating extensive involvement of AS in Wilms’ tumor pathogenesis. To prioritize dominant spliced transcripts (accounting for a larger proportion of total gene expression), we filtered differential variable splicing events by pSAR. Events with pSAR < 50% were excluded, retaining 1,669 events with pSAR ≥ 50% for subsequent analysis (Fig S1E in S1 File). PCA of splicing ratios for these events showed clear separation of the tumor and normal control groups by the first principal component (Fig S1F in S1 File). GO biological process (GO-BP) analysis revealed that the regulated alternative splicing genes (RASGs) were mainly enriched in broad, fundamental biological pathways such as non-membrane-bounded organelle assembly, small GTPase-mediated signal transduction, regulation of neuronal projection development, and regulation of cell morphogenesis (Fig S1G in S1 File), reflecting a global dysregulation of cellular architecture and signaling.

To further explore the specific roles of AS in cell death-related genes within Wilms’ tumor, we focused on a targeted sub-network of cell death-related RASEs between the two groups. By intersecting the genes associated with the aforementioned 1,669 RASEs (1,008 genes) with known cell death-related genes (2,009 genes), we obtained 118 overlapping genes (Fig 1B), which may play a crucial role in the regulation of cell death processes in Wilms’ tumor cells. SUVA analysis revealed a large number of cell death-related RASEs between the two groups, with alt5p and alt3p being the predominant types (Fig 1C). The splicing events were corresponding to classical splicing events, in which A5SS and ES events accounted for a large proportion (Fig 1D). GO-BP analysis specifically restricted to these 118 overlapping genes was performed to delineate the exact sub-pathways affected. This analysis showed significant enrichment of the cell death-related RASGs in the intrinsic apoptotic signaling pathway (Fig 1E), suggesting that within the broader splicing landscape, these cell death-related RASEs may primarily participate in the pathological process of Wilms’ tumor regulating apoptosis. The complete details of these RASEs, including their overlap with the 118 cell death-related genes, are provided in S1 Table. And the complete list of enriched GO terms and their corresponding mapped genes for all network analyses (Fig 1E, S1F, and 2F Fig in S1 File) is provided in S2 Table. These findings provide a new perspective for understanding the pathogenesis of Wilms’ tumor and lay the foundation for identifying potential therapeutic targets and biomarkers.

### Analysis of AS ratios and correlation with gene expression of cell death-related genes in Wilms’ tumor

**Fig 2:**
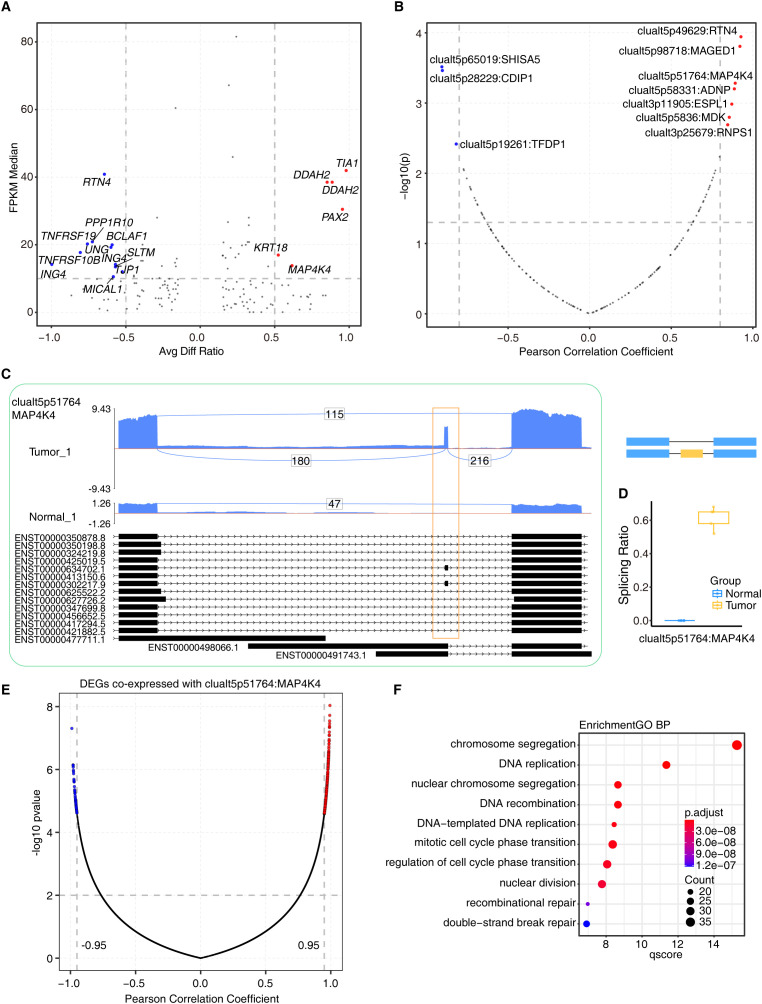
Correlation analysis of RASEs and gene expression. (A) Scatter plot illustrating the relationship between the differential splicing ratio of RASEs (x-axis) and the median expression of their host genes (y-axis). Host genes with a differential splicing ratio > 0.5 and a median FPKM > 10 are highlighted in red, while those with a ratio < −0.5 and median FPKM > 10 are highlighted in blue. (B) Volcano plot showing the Pearson correlation between RAS ratio and host gene expression. The x-axis represents the correlation coefficient, and the y-axis represents the significance (-log10 P-value). (C) Sogen plot showing the read distribution and genomic structure of the splicing event clualt5p51764: *MAP4K4*. (D) Box plot displaying the splicing ratio of the splicing event clualt5p51764: *MAP4K4* across samples. (E) Volcano plot showing the correlation between the splicing ratio of the event clualt5p51764: *MAP4K4* and the expression of DEGs. The x-axis represents the Pearson correlation coefficient, and the y-axis represents the P-value. DEGs with a correlation coefficient > 0.95 or <−0.95 are highlighted in red and blue, respectively. (F) Dot plot of the GO-BP enrichment analysis of DEGs that are highly correlated with the splicing event clualt5p51764: *MAP4K4*.

We analyzed AS ratio changes of cell death-related genes and their correlation with gene expression in Wilms’ tumor. Compared with the Normal group, the Tumor group showed a significantly increased splicing ratio of *MAP4K4*, and its expression level was significantly positively correlated with the splicing event proportion (Fig 2A-B), indicating that MAP4K4’s abnormal AS was accompanied by elevated expression, which may synergistically enhance its pro-tumor function. We visualized the read distribution of the splicing event clualt5p51764: *MAP4K4*, showing the Tumor group preferred the proximal 5’ splice site (resulting in exon retention) and had an increased splicing ratio (Fig 2C-D). To further verify the tumor specificity of this finding and control for inter-individual heterogeneity, we retrospectively examined this event using a strictly paired approach. The paired analysis revealed that the clualt5p51764:MAP4K4 splicing shift was consistently and significantly dysregulated in 4 out of the 5 matched patient pairs, confirming it as a robust, tumor-specific alteration rather than a subset-driven artifact. This indicates that the splicing pattern of MAP4K4 has high specificity and potential application value.

MAP4K4 is known to inhibit tumor cell apoptosis and promote proliferation [26]; its simultaneous upregulation of expression and abnormal AS ratio in Wilms’ tumor may contribute to a stronger pro-tumor effect via the combined action of expression enhancement and splicing pattern alteration. Given the importance of apoptotic pathway abnormalities in Wilms’ tumor [17], we focused on *MAP4K4*’s AS and expression changes. Correlation analysis between group-specific DEGs and clualt5p51764: *MAP4K4* identified significantly correlated DEGs (Fig 2E). Notably, the upstream splicing factor *HNRNPD* was found to be among the top upregulated DEGs exhibiting a strong, significant positive correlation with this *MAP4K4* splicing event, reflecting a potential regulatory crosstalk between gene expression and AS. These DEGs may be downstream effectors of *MAP4K4*’s abnormal AS, and their expression changes could amplify the impact on tumor biological processes. Together, the prominent presence of monocyte-recruiting DAMPs (HMGB1) and immune-modulatory molecules (HLA-E, FGL2, TGFBR2) strongly suggests that *MAP4K4* splicing-driven cell death may actively trigger immune cell recruitment.

GO-BP analysis showed these DEGs were enriched in chromosome separation, DNA replication, DNA recombination, cell cycle transition, and double-strand break repair (Fig 2F). While primarily driving intrinsic tumor proliferation and genomic instability, such pathways are increasingly recognized as triggers for TME remodeling via senescence-associated secretory phenotypes or inflammatory signaling. This indicates *MAP4K4*’s abnormal AS may affect tumor cell proliferation and genomic stability while simultaneously orchestrating the immune microenvironment by regulating these DEGs’ expression. A comprehensive list of all DEGs, including their expression metrics, overlap with splicing factors, and specific correlation coefficients with clualt5p51764:MAP4K4, is provided in S3 Table. Additionally, in tumors, *PRR5* preferred the distal 5’ splice site, with its splicing ratio (clualt5p62577: *PRR5*) decreased (Fig S2A in S1 File); *ROBO2* preferred the proximal 5’ splice site, with its splicing ratio (clualt5p66383: *ROBO2*) significantly upregulated (Fig S2B in S1 File).

### Construction of the SFs-RASEs-GO-BP terms regulatory network in Wilms’ tumor

**Fig 3:**
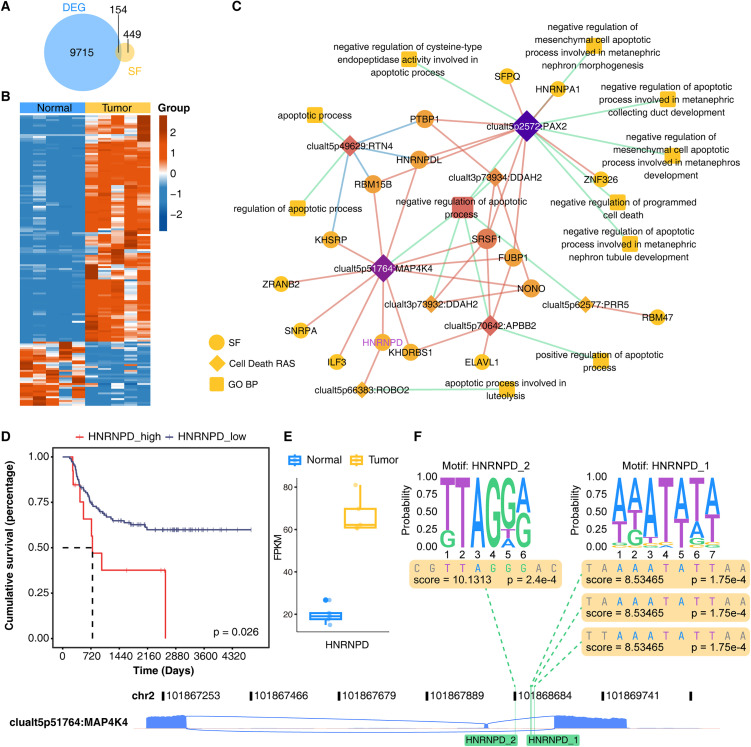
Regulatory network of SFs and cell death-related RASEs. (A) Venn diagram showing the number of DESFs. (B) Heatmap displaying the expression levels of the DESFs across samples. (C) The SFs-RAS regulatory network. Diamonds represent cell death-related RASEs, circles represent SFs, and rounded rectangles represent GO-BP terms. Red and blue edges indicate positive and negative correlations, respectively. Green edges link RASEs to the GO-BP terms of their host genes. The network was constructed using RASEs with |differential splicing ratio| > 0.5 and host gene FPKM > 3, and Spearman correlations with |R| > 0.95 and P < 0.01. (D) Kaplan-Meier survival analysis for *HNRNPD* based on data from the TARGET-WT cohort. (E) Box plot showing the expression of *HNRNPD*. (F) The predicted binding motif of the splicing factor *HNRNPD* on the splicing event clualt5p51764: *MAP4K4* in the *MAP4K4* gene, as calculated by RBPBench. Note that the HNRNPD_1 motif model exhibits three distinct predicted binding occurrences (hits) clustered closely within this genomic region, which are displayed individually.

To clarify the mechanism of cell death-related AS in Wilms’ tumor, we analyzed DESFs. First, a total of 9,869 DEGs were identified across the two groups of samples. Compared with the Normal group, 4,340 genes were upregulated and 5,529 genes were downregulated in the Tumor group (Fig S3A in S1 File); this widespread gene expression dysregulation provides a molecular basis for abnormal AS of cell death-related genes. Overlapping the DEGs with the 603 known SF genes yielded 154 DESFs (Fig 3A), and most DESFs were significantly upregulated in tumors (Fig 3B); their high expression is the core driver of the cell death-related genes’ abnormal AS. Further, we constructed a regulatory network of SFs-RASEs -GO-BP pathways, with relationships mainly involving apoptosis-related pathways. A total of 11 potential SFs were found to be positively correlated with the AS ratio of the splicing event clualt5p51764: *MAP4K4* (Fig 3C), implying they may be involved in its regulatory process. Furthermore, prognostic analysis using TARGET-WT data showed high expression of SF genes *ZRANB2*, *KHDRBS1*, *KHSRP*, *FUBP1* and *HNRNPD* correlated with poor prognosis (Fig 3D, Fig S3C in S1 File), and these SFs were highly expressed in tumors (Fig 3E, Fig S3B in S1 File). Finally, motif analysis predicted the binding sites of these 5 SFs on the pre-mRNA of *MAP4K4*. *HNRNPD* has two potential binding sites on the pre-mRNA of *MAP4K4*, with sequences TAAAATATTAA and CGTTAGGGAC, located at chr2 101868684–101869741 (Fig 3F). The specific binding sequence of *ZRANB2* on pre-mRNA was ATTGGTATCG, the specific binding sequence of *KHDRBS1* on pre-mRNA was CCTTTTTTCT, located at chr2 101867621–101867801. And the specific binding sequence of *KHSRP* on pre-mRNA was CACAGCCTCCTG, the specific binding sequence of *FUBP1* on pre-mRNA was TCTGTGTATAT, located at chr2 101867978–101869244 (Fig 3E). These sites support direct AS regulation, and SFs’ high expression ensures efficient pre-mRNA binding to promote abnormal AS.

### Association analysis between cell death-related AS and immune cell infiltration in Wilms’ tumor

Our central hypothesis postulates that intrinsic alternative splicing (AS) dysregulation primarily triggers abnormal cell death, which subsequently remodels the tumor microenvironment (TME). However, to provide a comprehensive complementary perspective, we first investigated whether immune-related genes themselves underwent direct splicing alterations. By intersecting our global RASEs dataset with a curated list of immune-related genes obtained from the ImmPort database, we identified a specific subset of immune-associated genes undergoing significant differential alternative splicing in Wilms’ tumor (Fig S4A in S1 File). This indicates that the intrinsic splicing dysregulation in Wilms’ tumor may also directly impact immune-related components.

**Fig 4:**
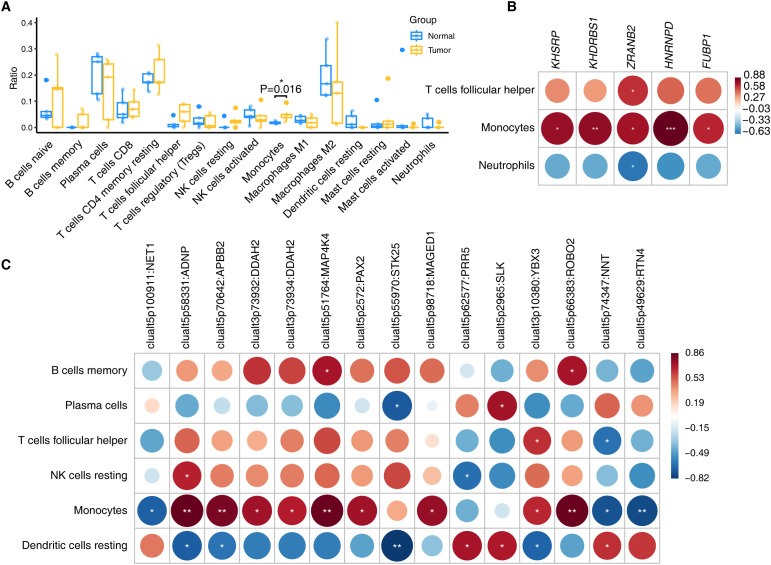
Correlation of RASEs and SFs with immune cell infiltration. (A) Boxplots showing the quantification of immune cell infiltration as determined by CIBERSORT. Statistical significance between the Tumor and Normal groups was evaluated across all 22 immune cell types using the Wilcoxon rank-sum test. Exact P-values for all cell types are available in S5 Table. (*P < 0.05). (B) Correlation heatmap illustrating the relationship between prognostically significant SFs and the abundance of infiltrating immune cells. Correlations were calculated using Spearman’s method (*P < 0.05, **P < 0.01, ***P < 0.001). (C) Correlation heatmap showing the association between the RASEs from the SFs-Cell death regulatory network (shown in Fig. 3C) and the levels of infiltrating immune cells. Correlations were calculated using Spearman’s method (*P < 0.05, **P < 0.01, ***P < 0.001).

To further evaluate the downstream macro-environmental shifts at the cellular level, we performed immune infiltration quantitative analysis using Cibersort. We found that only monocytes were significantly different between the two groups, with a significantly higher proportion of monocytes in tumors (Fig 4A). Correlation analysis between the SFs upstream of the *MAP4K4* splicing event and immune cell proportions showed that the expression of the prognostically significant SFs—*KHSRP*, *KHDRBS1*, *ZRANB2*, *FUBP1*, and notably *HNRNPD*—all exhibited strong positive correlations with the abundance of monocytes (Fig 4B). These SFs’ high expression may explain increased monocytes by modulating immune cell infiltration, linking AS regulation to immune microenvironment composition. Combined with the results of the previous prognostic analysis, we observed that the splicing events clualt5p66383: *ROBO2* and clualt5p51764: *MAP4K4*, which are potentially regulated by the splicing factor HNRNPD, all showed a significant positive correlation with the proportion of monocytes (Fig 4C). From this, it can be preliminarily inferred that this regulatory effect, on the one hand, can impact the apoptotic process of tumor cells (contributing to apoptosis resistance). On the other hand, it can indirectly modulate the infiltration of monocytes by releasing signaling molecules, thereby participating in the regulation of the immune microenvironment.

### Verification of the potential regulatory effect of HNRNPD on the AS of MAP4K4 using HNRNPD knockout datasets

**Fig 5:**
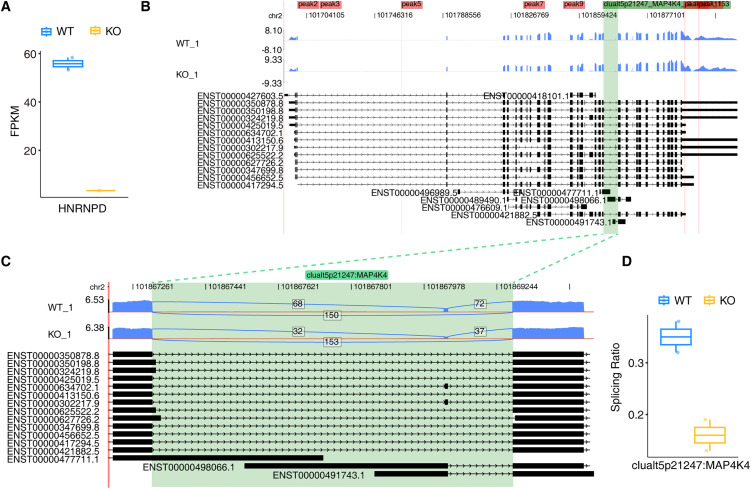
Validation of HNRNPD splicing regulation using knockout data. (A) Boxplot showing the expression of *HNRNPD* in the knockout dataset. (B) Sogen plot displaying the mRNA-seq read distribution (WT vs. KO) across the *MAP4K4* gene locus. Binding peaks identified from HNRNPD FLASH-seq data (GSE212767) are highlighted as peak tracks in red to indicate direct interaction sites. (C) Sogen plot illustrating the detailed splicing structure of the splicing event clualt5p51764: *MAP4K4* following *HNRNPD* knockout. (D) Boxplot comparing the splicing ratio of the event clualt5p51764: *MAP4K4* between *HNRNPD* knockout and control samples.

For HNRNPD, we downloaded and analyzed the transcriptomic dataset GSE212767 of *HNRNPD* knockout (KO) and control (WT) HEK-293T cells to verify its potential regulatory effect on the AS of *MAP4K4*. First, we confirmed *HNRNPD* expression was significantly lower in the KO group than in the WT group (Fig 5A). By integrating the corresponding HNRNPD FLASH-seq data, we identified direct protein-RNA binding peak tracks clustered within the genomic region of MAP4K4. Analysis revealed that in the KO group, *MAP4K4* tended to select the distal 5’ splice site (Fig 5B, C), and the splicing ratio of clualt5p21247: *MAP4K4* was significantly reduced (Fig 5D). This provides data support for the potential regulatory role of HNRNPD in the AS of *MAP4K4*.

## Discussion

This study provides preliminary evidence that the abnormal expression of SFs represented by HNRNPD drives the abnormal AS of the cell death-related genes such as *MAP4K4*, *PRR5*, and *ROBO2*. The combined effects of SF overexpression, target gene AS abnormalities, and corresponding changes in target gene expression levels support the potential existence of an “SF-cell death-related AS-immune microenvironment” regulatory axis, which may jointly promote Wilms’ tumor development. These findings not only clarifies the key molecular mechanism connecting AS, apoptosis, and immune microenvironment in Wilms’ tumor but also provides potential targets for the development of prognostic biomarkers and splicing-targeted therapies—though further functional experiments are required to confirm the causality and clinical relevance of this axis.

AS analysis revealed significant differences in the patterns of cell death-related RASEs between the Tumor and Normal groups. It is noteworthy that in the global functional enrichment analysis of all 1,008 RASGs (Fig S1F in S1 File), canonical cell death terms were not ranked among the top hits. This is because global splicing aberrations in Wilms’ tumor preferentially affect broad, fundamental cellular machinery (such as organelle assembly and intracellular signaling), which statistically eclipse more targeted sub-processes in a genome-wide hypergeometric test. However, when specifically isolating the 118 overlapping cell death genes, the functions of these cell death-related RASGs were mainly enriched in the apoptotic signaling pathway, further confirming that apoptosis is the core pathway of cell death in Wilms’ tumor. In-depth analysis showed obvious abnormalities in AS of genes such as *MAP4K4*, *PRR5*, and *ROBO2* in the Tumor group. Among them, the *PRR5* gene encodes a proline-rich protein with a complex expression pattern, generating multiple mRNA variants through alternative promoters and AS [27]. PRR5 is one of the key components of the mTORC2 complex, which plays an important role in cell growth, survival, and metabolism [28]; its silencing inhibits proliferation-related pathways, and deletions in its chromosomal region are associated with various tumors [28,29]. Regarding ROBO2, it is a single-pass transmembrane receptor belonging to the immunoglobulin superfamily and is involved in kidney development and angiogenesis [30,31]. In tumors, ROBO2 functions in a tissue-specific manner. Its expression is lost in pancreatic ductal adenocarcinoma, where it acts as a stromal suppressor by inhibiting myofibroblast activation and T-cell infiltration [32]; in glioblastoma, ROBO2 exerts a pro-tumor function by mediating the PI3K-γ signaling pathway (together with ROBO1) to drive the chemotaxis and polarization of tumor-associated microglia/macrophages, which in turn induces abnormal tumor vasculature, causes immune suppression, and ultimately promotes tumor growth [33]; in hepatocellular carcinoma, ROBO2 is highly expressed, and its downregulation inhibit the EMT and proliferation of liver cancer cells and accelerate apoptosis [34]. Specifically, MAP4K4 is a Ser/Thr kinase that regulates cell proliferation, survival, cytoskeleton dynamics, and ion transport. Overexpressed in tumors (e.g., colorectal, liver, gastric, lung, prostate, pancreatic cancers), it correlates with progression, metastasis, and poor prognosis, promoting tumor cell proliferation/migration/invasion via JNK signaling and exerting anti-apoptotic effects [26]. In hepatocellular carcinoma and pancreatic cancer, inhibiting/downregulating MAP4K4 reduces tumor growth, increases apoptosis, and improves chemosensitivity, confirming its inhibition suppresses tumors by promoting apoptosis and inhibiting proliferation [35,36]. It also modulates T cell-mediated immunity, with its dysfunction impairing anti-tumor and antiviral responses [26]; and, in atherosclerosis, drives immune cell (e.g., monocyte) recruitment via inflammatory/chemokine pathways—its absence/inhibition lessens atherosclerotic lesions [37]. At present, no direct association has been found between PRR5, ROBO2, and MAP4K4 and Wilms’ tumor. Given the close relationship between MAP4K4 and tumors as well as apoptosis, we conducted further analysis of its AS event clualt5p51764: *MAP4K4*. At the protein level, canonical alternative splicing of MAP4K4 primarily alters its intrinsically disordered interdomain linker region while preserving the core catalytic kinase and regulatory CNH domains. Literature indicates that such variations within the linker region modify structural flexibility and protein-protein interaction capabilities, directly impacting downstream c-Jun N-terminal kinase (JNK) phosphorylation and signaling efficiency [26]. Therefore, the tumor-specific splicing shift of MAP4K4 predicted in our cohort may disrupt standard pathway kinetics, favoring anti-apoptotic signaling over standard regulatory loops, thereby affecting the proliferative capacity and genomic stability of tumor cells. We found that this splicing event may regulate related DEGs to participate in key biological processes in Wilms’ tumor, such as chromosome separation, DNA replication, and cell cycle transition, thereby affecting the proliferative capacity and genomic stability of tumor cells.

Based on SF and prognostic analyses, we identified ZRANB2, KHDRBS1, KHSRP, FUBP1, and HNRNPD as potential SFs that may mediate cell apoptosis. Additionally, they all have potential binding sites on the pre-mRNA of *MAP4K4*, suggesting their potential involvement in regulating the AS of *MAP4K4*. Apoptosis, also known as programmed cell death, is a critical process that helps maintain normal physiological conditions, control disease progression, and preserve tissue structure and function [38,39]. Dysregulation of apoptosis in tumor cells can lead to abnormal cell growth and survival. Regarding ZRANB2, it exerts oncogenic effects in various tumors. It is upregulated in glioma, and its knockdown inhibits glioma cell proliferation and the formation of angiogenesis-mimicking tube-like structures [40]. It is also upregulated in hepatocellular carcinoma, where its knockdown suppresses tumor cell proliferation and promotes apoptosis [41]. *KHDRBS1* encodes the RNA-binding protein Sam68, an oncogenic SF involved in signal transduction and pre-mRNA splicing, regulating mRNA export and stability, cell death, mitosis, and cell cycle progression [42]. It controls the AS of many pre-mRNAs encoding oncoproteins, such as cyclin D1, CD44, and Bcl-x [43]. KHSRP is associated with tumor progression in different types of malignancies. It interacts with SF3B1 to facilitate mRNA splicing for genes like *EGFR* and *CDC25A*, maintaining functional protein expression, thereby enhancing hepatocyte proliferation and inhibiting apoptosis, which is protective in acute liver failure [44]. KHSRP is highly expressed in Wilms’ tumor and is associated with poor prognosis. It binds to the 3’UTR of *PPP2CA* mRNA and regulates its mRNA stability, thereby modulating the phosphorylation levels and protein stability of p27 in tumor cells, influencing cell proliferation and cell cycle [45]. FUBP1 is a single-stranded nucleic acid-binding protein that regulates DNA transcription, RNA biogenesis, and translation by binding to DNA and RNA [46]. It has been identified as a potent pro-proliferative and anti-apoptotic factor, which is abnormally overexpressed in a variety of human cancers [46,47]. Mechanistic studies have found that multiple regions of *NORAD* can bind to the central domain of the anti-apoptotic factor *FUBP1*, weakening its nuclear localization and thereby affecting FUBP1’s occupancy of the promoters of its target pro-apoptotic genes, inhibiting their expression and inducing apoptosis in endometrial cancer cells [48]. HNRNPD can modulate the stability of mRNAs encoded by genes involved in tumorigenesis, cell cycle, senescence, proliferation, and apoptosis [49]. In type 1 diabetes (T1D), overexpression of HNRNPD leads to pancreatic β-cell apoptosis by downregulating the expression of anti-apoptotic proteins BCL2 and MCL1. Conversely, silencing of HNRNPD can restore anti-apoptotic proteins and attenuate the NF-κB transcription factor, thereby promoting the survival of pancreatic β-cells [50]. In oral squamous cell carcinoma, as a downstream target gene of NF-κB, overexpression of HNRNPD can stabilize anti-apoptotic molecule mRNAs and degrade pro-apoptotic molecule mRNAs, thereby helping to maintain the apoptosis-resistant state of tumor cells and promoting tumor progression [51]. In clear cell renal cell carcinoma, HNRNPD inhibits tumor cell apoptosis by regulating the RNA splicing balance of key genes (centered on the mRNA to circRNA ratio of CDK1), becoming an important molecular mechanism for tumor cells to evade apoptosis and maintain a malignant phenotype [49]. In Wilms’ tumor, HNRNPD has been identified as one of the tumor-associated DEGs and is a core gene in the protein-protein interaction network [52], although its specific role in Wilms’ tumor is not yet fully understood. Combining the analysis of *HNRNPD* knockout data, our study shows that HNRNPD may be involved in the AS of *MAP4K4* in Wilms’ tumor.

Through Cibersort-based quantification of immune infiltration, we found that monocytes were the only immune cell type with a significantly different proportion between tumor and normal groups, and this proportion was notably higher in tumors. This finding provides a crucial clue for understanding the immune microenvironment characteristics of Wilms’ tumor. Monocytes serve as a bridge connecting innate and adaptive immune responses and have multiple biological roles in tumor immunity [53]. It is worth noting that previous study reported more extensive immune remodeling in tumors, including increased CD4 memory T cells and decreased plasma cells, CD8 T cells, and Tregs [17]. In contrast, our analysis identified monocytes as the only significantly altered population. This discrepancy is highly likely attributable to the limited statistical power inherent to our small discovery cohort (GSE138869, n = 5 pairs), which restricts our ability to detect subtle cellular shifts but highlights monocytes as a highly robust change in these samples. Monocyte infiltration has a protective effect, with its level being positively correlated with patient prognosis [54]. However, monocytes can also differentiate into tumor-associated macrophages (TAMs), which promote tumor cell migration and invasion, thereby driving tumor progression, through the secretion of molecules such as MMP9 and via pathways like NF-κB [55]. Overall, monocytes influence the progression of Wilms’ tumor through their infiltration and the functions of TAMs after differentiation [54]. In this study, the increased proportion of monocytes in tumors may reflect a potential trend toward differentiation into TAMs, thereby contributing to the construction of an immunosuppressive microenvironment. This finding provides new cellular evidence for elucidating the immune escape mechanisms of Wilms’ tumor.

The strong correlation between cell death-related RASEs, SFs, and monocytes may indicate that AS serves as a core molecular hub for “cell death regulation” and “immune microenvironment remodeling” in Wilms’ tumor. On the one hand, apoptosis imbalance regulates immune infiltration through signaling pathways. In tumor cells, abnormal AS of *MAP4K4* potentially regulated by the splicing factor HNRNPD may lead to apoptosis resistance. These tumor cells may enhance their ability to recruit monocytes by secreting chemokines or abnormally expressing surface signaling molecules, resulting in an increased proportion of monocytes in the tumor tissue. Monocytes further differentiate into M2 TAMs, which construct an immunosuppressive microenvironment by secreting immune-suppressive cytokines [55,56]. On the other hand, immune infiltration affects the apoptosis process through feedback regulation. In the immunosuppressive microenvironment, cytokines secreted by M2 macrophages can activate anti-apoptotic pathways in tumor cells, forming a vicious cycle of “apoptosis resistance—monocyte enrichment—immunosuppression—more significant apoptosis resistance”. Ultimately, these factors collectively promote the development and progression of Wilms’ tumor.

## Conclusion

In conclusion, our study demonstrates that the apoptosis imbalance caused by the aberrant AS of apoptosis-related genes regulated by SFs, together with the abnormal immune microenvironment, may jointly promote the development of Wilms’ tumor. Specifically, SFs regulate the cell death-related RASEs, which not only directly determine the apoptotic fate of tumor cells (e.g., acquisition of anti-apoptotic phenotypes) but also indirectly regulate the recruitment, infiltration, and functional differentiation of immune cells (especially monocytes) through downstream signal changes triggered by apoptotic imbalance. This forms a coordinated regulatory network of “AS-apoptosis-immunity”, which collectively drives the pathological progression of tumors. For instance, the splicing factor HNRNPD may enhance the apoptotic resistance of tumor cells by regulating the alt5p-type AS of *MAP4K4*, and its expression level is also significantly correlated with the proportion of monocytes. This result suggests a potential key mediating role of AS in connecting apoptosis and immunity.

However, this study still has certain limitations. Firstly, dataset selection and sequencing depth present inherent constraints. Precise alternative splicing quantification using the SUVA algorithm strictly requires raw sequencing data (FASTQ/BAM). Due to controlled-access restrictions on the TARGET-WT raw data, we utilized its public expression matrix for survival analysis and relied on GSE138869 for splicing profiling. Furthermore, the sequencing depth of GSE138869 is relatively low (<25 million reads per sample); while sufficient to identify highly robust splicing alterations like MAP4K4, it may miss low-abundance variants. Secondly, as a strictly in silico and computational study, our findings rely on transcriptomic analysis and cross-cohort validation. There is a lack of direct protein-level validation in clinical tissues, and the exact physical molecular interactions governing SF-mediated AS regulation remain to be deeply explored. Thirdly, the biological impact of the “AS-apoptosis-immune” regulatory axis on Wilms’ tumor growth has not been verified through in vivo models, which makes it difficult to fully reflect the actual role of this regulatory network in physiological and pathological environments. Based on these limitations, our subsequent research plan is as follows. At the cellular level, by constructing Wilms’ tumor cell lines with HNRNPD knockout/knockin, the direct binding and regulatory mechanism between HNRNPD and the precursor mRNA of the target gene MAP4K4 will be experimentally clarified. At the animal level, a nude mouse xenograft model of Wilms’ tumor will be established, and through tail vein injection of SF-interfering lentiviruses or splicing-targeting small molecule drugs, the clinical translational value of key SFs and RASEs in Wilms’ tumor will be further verified.

## Supporting information

S1 File Supplementary figures. Combined Supporting Information Figures. This document contains supplementary figures S1–S4.
(DOCX)

S1 Table Comprehensive list of Regulated Alternative Splicing Events (RASEs) identified in Wilms’ tumor and adjacent normal tissues. (XLSX)

S2 Table Significantly enriched Gene Ontology (GO) terms and associated gene lists. (XLSX)

S3 Table Profiling of differentially expressed genes (DEGs) in Wilms’ tumor, including splicing factor (SF) annotation and co-expression analysis with clualt5p51764:MAP4K4. (XLSX)

S4 Table Comprehensive catalog of 603 human splicing factor genes. (XLSX)

S5 Table Statistical analysis of immune cell infiltration differences between Wilms’ tumor and normal tissues. (XLSX)
