# Supplementary material for: Splicing factor HNRNPD and alternative splicing of MAP4K4 are associated with cell apoptosis and immune microenvironment features in Wilms’ tumor
Source: PLoS One. 2026 Aug 6;21(8):e0354712. doi: 10.1371/journal.pone.0354712 (PMC13446680; doi:10.1371/journal.pone.0354712)
Supplement: Supplementary file 1 [file pone.0354712.s001.docx]

**Figure S1. Alternative Splicing Analysis using SUVA.**

(A) Schematic diagram illustrating the five types of alternative splicing events defined by SUVA.

(B) Box plot showing the number of all AS event types detected by SUVA.

(C) The number of SUVA-detected alternative splicing events corresponding to classic AS types.

(D) Bar plot showing the distribution of pSAR (Proportion of SUVA AS Events Ratio); AS events with pSAR > 50% were selected for downstream analysis.

(E) Principal Component Analysis (PCA) based on the splicing ratios of SUVA alternative splicing events.

(F) Gene Ontology (GO) enrichment analysis for Biological Processes (BP) of the host genes of RAS events.

**Figure S2. Visualization of Additional Alternative Splicing Events.**

(A-B) Sogen plots (left) show the read distribution and alternative splicing structure for events clualt5p62577 and clualt5p66383. The boxplots on the right show the corresponding splicing ratio for each event.

**Figure S3. Analysis of Key Splicing Factors and their Potential Targets.**

(A) Volcano plot showing the differentially expressed genes between tumor and normal tissues (DESeq2, FDR < 0.05 and |logFC| > 1).

(B) Boxplots displaying the expression levels of four splicing factors: ZRANB2, KHDRBS1, KHSRP, and FUBP1.

(C) Kaplan-Meier survival analysis for the four splicing factors: ZRANB2, KHDRBS1, KHSRP, and FUBP1.

(D) Predicted binding motifs for ZRANB2, KHDRBS1, KHSRP, and FUBP1 on the splicing event clualt5p51764, as determined by RBPBench.

**Figure S4. Alternative splicing of immune-related genes in Wilms' tumor.** (A) Heatmap displaying the differential alternative splicing events of immune-associated genes (curated from the ImmPort database) between Wilms' tumor and matched adjacent normal tissues.
